# Supplementary material for: Mind training, stress and behaviour—A randomised experiment
Source: PLoS One. 2021 Nov 12;16(11):e0258172. doi: 10.1371/journal.pone.0258172 (PMC8589216; doi:10.1371/journal.pone.0258172)
Supplement: S1 File — (PDF) [file pone.0258172.s001.pdf]

Supplementary Materials

Appendix A: Recruitment Poster and Email

Participate in a LIFESTYLE study!

The School of Economics at the University of Edinburgh is currently seeking

150 HEALTHY individuals

Do you sometimes feel stressed? Want to participate in a scientific study and earn a bit of money?

Are you available for a couple of hours a week (at a time of your choice) between mid-October and mid-November?

GOAL OF THE STUDY

The goal of this study is to look at stress and lifestyle among university students. If you decide to participate, you will be given a specific protocol that you will be kindly requested to follow for four consecutive weeks. These protocols are non-invasive and will include requests to undertake certain activities during the week, for a period of four weeks starting immediately after the initial session.

Note: you must be **at least 18 years old,** a **student** at the University of Edinburgh, and have **NO pre-existing medical conditions**

INTERESTED? WANT MORE INFORMATION?

E-mail: blue@ed.ac.uk

FEELING A BIT STRESSED? WANT TO EARN SOME MONEY? HAVE A COUPLE OF HOURS PER WEEK TO SPARE IN THE COMING TWO MONTHS?

Participate to our study on "Stress and Lifestyle among University Students".

The Behavioural Laboratory at the University of Edinburgh is currently seeking <u>150 HEALTHY individuals</u> for a scientific study on stress and lifestyle among university students.

- **You will be asked to come every week to our laboratory at a specific timeslot (the same day and same time every week) <u>for a period of 6 weeks</u> (starting in the week of October 22d) and another time 4 months later (in March 2015).**
- **You will be asked to follow <u>a specific protocol</u> in between (more information below).**

Please read on before signing up.

DESCRIPTION OF THE STUDY

The goal of this study is to look at stress and lifestyle among university students. If you decide to participate, you will be given a **<u>specific protocol</u>** that you will be kindly requested to follow for **four consecutive weeks**. These protocols are non-invasive and will include requests to undertake certain activities during the week, for a period of four weeks starting immediately after the initial session. **It is very important for our study that you agree to follow the protocol's instructions**. These activities should not take more than 2 hours a week and **we will pay all costs involved**.

The study will take place over the course of 6 weeks and an additional follow-up in six months. You will be asked to come to our experimental laboratory (situated at the) every week (6 times in total including the 6 months follow up). You will be asked to come every week **<u>on the same day and same time slot (this is VERY important for our analysis, so please do pick your timeslot carefully and make sure you can come every week)</u>**.

Each time, you will be asked to answer basic survey questions (including basic background information), questions about your lifestyle and health, feedback on the protocol you have been asked to follow and you will be asked to take decisions that will involve monetary rewards (you can only earn positive amounts). **<u>You should expect to receive between £3 and £10 in each session (the exact amount will depend on your decisions).</u>** The sessions in weeks 1 and 6 (and in March 2015) will last about an hour and a half. The other sessions will take less than half an hour each.

Note that in sessions 1 and 6, we will collect saliva samples using a standard scientific protocol. The protocol is non invasive and completely safe. The goal is to measure cortisol concentration (as an indicator of stress levels). <u>We kindly ask you not to drink or eat anything one hour prior to the session</u>.

<u>Eligibility criteria:</u>

- You must be older than 18 years old, student at the University of Edinburgh, with NO medical condition

Possible time slot options (you can only choose one option and are asked to stick to the days and times once you have picked that option)

	Option 1	Option 2	Option 3	Option 4	Option 5	Option 6
Week 1<br>Initial session<br>(1 hour 30 min)<br>Pay: £3-10	Tue 21/10<br>10 am	Tue 21/10<br>12.30 pm	Tue 21/10<br>3 pm	Wed 22/10<br>10 am	Wed 22/10<br>12.30 pm	Wed 22/10<br>3.30 pm
Week 2<br>(30 min)<br>Pay: £7.50	Tue 28/10<br>10 am	Tue 28/10<br>12.30 pm	Tue 28/10<br>3 pm	Wed 29/10<br>10 am	Wed 29/10<br>12.30 pm	Wed 29/10<br>3.30 pm
Week 3<br>(30 min)<br>Pay: £7.50	Tue 4/11<br>10 am	Tue 4/11<br>12.30 pm	Tue 4/11<br>3 pm	Wed 5/11<br>10 am	Wed 5/11<br>12.30 pm	Wed 5/11<br>3.30 pm
Week 4<br>(30 min)<br>Pay: £7.50	Tue 11/11<br>10 am	Tue 11/11<br>12.30 pm	Tue 11/11<br>3 pm	Wed 12/11<br>10 am	Wed 12/11<br>12.30 pm	Wed 12/11<br>3.30 pm
Week 5<br>(30 min)<br>Pay: £7.50	Tue 18/11<br>10 am	Tue 18/11<br>12.30 pm	Tue 18/11<br>3 pm	Wed 19/11<br>10 am	Wed 19/11<br>12.30 pm	Wed 19/11<br>3.30 pm
Week 6<br>(1 hour 30 min)<br>Pay: £3-£10	Tue 25/11<br>10 am	Tue 25/11<br>12.30 pm	Tue 25/11<br>3 pm	Wed 26/11<br>10 am	Wed 26/11<br>12.30 pm	Wed 26/11<br>3.30 pm
4 months later<br>(1 hour 30 min)<br>Pay: £3-£10	Tue 18/3<br>10 am	Tue 18/3<br>12.30 pm	Tue 18/3<br>3 pm	Wed 19/3<br>10 am	Wed 19/3<br>12.30 pm	Wed 19/3<br>3.30 pm

Location of the sessions: Behavioural Laboratory at the University of Edinburgh, School of Economics, 31 Buccleuch Place, 4th floor

Ethical issues and Informed consent

Note that the study is conducted with ethical approval of the School of Economics at the University of Edinburgh. All the data will be anonymised and treated with confidentiality, in accordance with the ethical guidelines.

You will be asked to sign an informed consent form at the beginning of the initial session. Although we emphasize the importance of participating to all sessions, you will be free to withdraw from the study at any point in time.

Interested? Please e-mail blue@ed.ac.auk and indicate which option you would prefer.

Appendix B: Evaluation of the Treatment and Control Intervention for Relaxation Purposes

How useful was the program (control: BBC Ancient Worlds; treatment: Mindfulness) for relaxation purposes? 1-very useful, 2-somewhat useful, 3-not useful at all.

Session		Control, previous week	Control, overall	Treatment, previous week	Treatment, overall
2	mean (sd)	2.00 (0.54)		2.05 (0.57)	
	median	2		2	
3	mean (sd)	2.10 (0.65)		1.92 (0.65)	
	median	2		2	
4	mean (sd)	1.97 (0.54)		1.97 (0.60)	
	median	2		2	
5	mean (sd)	2.03 (0.57)		1.86 (0.52)	
	median	2		2	
6	mean (sd)	2.10 (0.61)	2.13 (0.55)	1.98 (0.51)	2.02 (0.51)
	median	2	2	2	2

Table 1: Evaluation of the Treatment and Control Intervention for Relaxation Purposes

Appendix C: Weekly Survey

Weekly survey

Q1 Please enter your desk number.

Q2 This survey aims to collect information on various aspects of your lifestyle and activities in the previous week. The survey takes around 30 minutes to complete. All data collected in this survey will be held anonymously and securely, and will be used for research purposes only. Please click next to continue.

Q3 Please enter your participant ID. This is the code consisting of letters and numbers on the card you were given at registration. Please don't put any spaces between the letters and numbers.

Q4 We would like to ask you some questions about your feelings on aspects of your life. There are no right or wrong answers. For each of these questions we'd like you to give an answer on a scale of 0 to 10, where 0 is 'not at all' and 10 is 'completely'.

	0 (1)	1 (2)	2 (3)	3 (4)	4 (5)	5 (6)	6 (7)	7 (8)	8 (9)	9 (10)	10 (11)
Overall, how satisfied were you with your life in the previous week? (1)	❍	❍	❍	❍	❍	❍	❍	❍	❍	❍	❍
Overall, to what extent do you feel that the things you did the previous week were worthwhile? (2)	❍	❍	❍	❍	❍	❍	❍	❍	❍	❍	❍
Overall, how happy are you these days? (3)	❍	❍	❍	❍	❍	❍	❍	❍	❍	❍	❍
Overall, how happy are you right now? (5)	❍	❍	❍	❍	❍	❍	❍	❍	❍	❍	❍
Overall, how anxious do	❍	❍	❍	❍	❍	❍	❍	❍	❍	❍	❍

you feel these days? (6)											
Overall, how anxious do you feel right now? (4)	❍	❍	❍	❍	❍	❍	❍	❍	❍	❍	❍

Q97 How many hours did you spend studying the previous week? Do NOT include hours spent in classes, but DO include hours spent studying alone, in the library or with classmates.

Q98 Did you have any midterm exams in the previous week?
❍ Yes (1)
❍ No (2)

Q99 Did you have to submit any assignments in the previous week?
❍ Yes (1)
❍ No (2)

Q100 Did anything unusually upsetting or stressful happen to you in the previous week?
❍ Yes (1)
❍ No (2)

Answer If Did anything unusually upsetting or stressful happen to you in the previous week? Yes Is Selected
Q101 Please provide some details.

Q5 The next questions are about your health behaviours during the PREVIOUS WEEK.

Q102 Please read all the following statements carefully and tick the box next to the one that best describes you.   During the previous week:
❍ I did not smoke any cigarette, not even a puff (1)
❍ I smoked cigarettes, but fewer than one per day (2)
❍ I smoked between 1 and 10 cigarettes per day (3)
❍ I smoked between 10 and 20 cigarettes per day (4)
❍ I smoked more than 20 cigarettes per day (5)

Q8 How often did you eat breakfast in the previous week?
❍ Almost every day (1)
❍ Most days a week (2)
❍ About once a week (3)
❍ Never (5)

Q9 How often did you eat lunch in the previous week?
- ❍ Almost every day (1)
- ❍ Most days a week (2)
- ❍ About once a week (3)
- ❍ Never (5)

Q10 How often did you eat dinner in the previous week?
- ❍ Almost every day (1)
- ❍ Most days a week (2)
- ❍ About once a week (3)
- ❍ Never (5)

Q11 Did you eat at regular times of the day during the previous week?
- ❍ Almost always (1)
- ❍ Most days (2)
- ❍ Sometimes (3)
- ❍ Never (4)

Q12 On average, how many meals did you eat each day during the previous week?

Q13 On average, how many between-meal snacks did you eat each day during the previous week?

Q16 These questions ask about what you ate or drank YESTERDAY. Tick all relevant boxes for each item (you can tick more than once as you could have the same type of meal for example for lunch and for dinner as well). There are no right or wrong answers. Did you eat (drink)…

	For breakfast (1)	For lunch (2)	For dinner (3)	Between main meals (4)	Not at all (5)
Processed meats like bacon, ham, sausage, or processed lunchmeats (1)	❑	❑	❑	❑	❑
Deep fried food, e.g. chips, onion rings, fried chicken, battered fish (2)	❑	❑	❑	❑	❑
Burgers, hot dog, pizza, sausage rolls (3)	❑	❑	❑	❑	❑
Potato crisps (4)	❑	❑	❑	❑	❑
Vegetable crisps (5)	❑	❑	❑	❑	❑
Popcorn, salted peanuts (6)	❑	❑	❑	❑	❑
Unsalted nuts, seeds (e.g. sunflower, pumpkin) (7)	❑	❑	❑	❑	❑
Pretzels, crackers, e.g. Ritz (8)	❑	❑	❑	❑	❑
Pies (savoury or sweet) (9)	❑	❑	❑	❑	❑
Cakes, muffins, brownies, cookies (10)	❑	❑	❑	❑	❑
Doughnut, pastry, e.g. Danish pastry, croissant, pain chocolat (11)	❑	❑	❑	❑	❑

Chocolate, candy bars, candies (12)	❑	❑	❑	❑	❑
Ice cream (13)	❑	❑	❑	❑	❑
Energy bar, high protein bar, e.g. Zone, PowerBar (14)	❑	❑	❑	❑	❑
Breakfast bar, e.g. Nutri-Grain (15)	❑	❑	❑	❑	❑
Soft drinks, e.g. Coke, Fanta, sugared sweetened fruit juices (16)	❑	❑	❑	❑	❑
Energy drinks, e.g. RedBull (17)	❑	❑	❑	❑	❑

Q17 How many servings of fruit did you eat yesterday? One serving is about one cup of chopped or sliced fruits, or one medium sized apple of banana.

Q18 Not counting potatoes, how many servings of vegetables did you eat yesterday? One serving is about one cup of chopped or sliced vegetables.

Q19 How often did you drink coffee, latte or cappuccino (not decaf) in the previous week?
❍ Never (1)
❍ About once a week (3)
❍ Every 2 or 3 days (4)
❍ Once a day (5)
❍ Twice a day (6)
❍ At least three times a day (7)

Q25 The next questions are about drinking alcohol, including beer, wine, spirits and any other alcoholic drink.

Q26 How many days over the previous week did you have an alcoholic drink?
- ❍ Almost every day (1)
- ❍ Most days a week (2)
- ❍ About once or twice (3)
- ❍ Never (5)

Q27 On the days that you did drink during the previous week, how many drinks did you have, on average? One drink is a glass of wine, or a pint of beer or cider, or 25 ml of spirits.

Q28 How often did each of the following happen to you during the previous week?

	All of the time (1)	Often (2)	Sometimes (3)	Rarely (4)	Never (5)
Felt completely out of control when it came to food. (1)	❍	❍	❍	❍	❍
Ate too much because you were upset, nervous or stressed. (2)	❍	❍	❍	❍	❍
Ate too much because you were bored or felt lonely. (3)	❍	❍	❍	❍	❍
Ate so much food so fast that you didn't know how much you ate or how it tasted. (4)	❍	❍	❍	❍	❍
Ate more than usual while preparing for an exam or working on an assignment. (5)	❍	❍	❍	❍	❍
Ate high calorie snacks while studying or working on assignments. (6)	❍	❍	❍	❍	❍

Q33 At what time did you go to sleep most days during the previous week?
❍ before 8pm (1)
❍ 8-9pm (2)
❍ 9-10pm (3)
❍ 10-11pm (4)
❍ 11pm-midnight (5)
❍ midnight-1am (6)
❍ 1am-2am (7)
❍ after 2am (8)

Q34 On average, how many hours of sleep did you get in a 24 hour period during the previous week?

Q35 How did you relax during the previous week?

	Almost every day (1)	Most days a week (2)	About once a week (3)	Never (5)
Watch movies / read books / listen to music (1)	❍	❍	❍	❍
Go to the cinema / theatre / concert (2)	❍	❍	❍	❍
Meet with friends (3)	❍	❍	❍	❍
Yoga / pilates / tai chi / chi gong or similar exercises (4)	❍	❍	❍	❍
Meditate / do breathing exercises / practice mindfulness (5)	❍	❍	❍	❍
Do sport activities (6)	❍	❍	❍	❍
Other (7)	❍	❍	❍	❍

Answer If How do you relax? Other - Almost every day Is Selected Or How do you relax? Other - About once or twice a week Is Selected Or How do you relax? Other - About once or twice a month Is Selected
Q36 What other activities did you do to relax, not listed above?

Q75 What time did you get up today?

Q76 What time did you go to sleep last night?

Q47 Please enter the code announced by the experimenters to continue.

Answer If Please enter the code announced by the experimenters to continue. Text Response Is Equal to AW37

Q103 Please summarize in at least 100 words the episode of the Ancient Worlds series you watched the previous week. You might add some of the following details: What were the main locations and topics? What did you learn from the documentary? Which parts did you find the most interesting or stunning?

Answer If Please enter the code announced by the experimenters to continue. Text Response Is Equal to AW37

Q104 Please provide a critical review of at least 50 words of the Ancient Worlds episode you watched the previous week. Feel free to add positive and negative remarks as well.

Answer If Please enter the code announced by the experimenters to continue. Text Response Is Equal to AW37

Q105 Did you find watching the Ancient Worlds episode useful for relaxation purposes?

- Very useful (1)
- Somewhat useful (2)
- Not useful at all (3)

Answer If Please enter the code announced by the experimenters to continue. Text Response Is Equal to AW37

Q106 Overall, how would you rate the Ancient Worlds episode you watched during the previous week?

- Excellent (1)
- Very good (2)
- Fair (3)
- Poor (4)

Answer If Please enter the code announced by the experimenters to continue. Text Response Is Equal to AW37

Q107 Would you recommend the Ancient Worlds documentary series to a friend?

- Yes (1)
- No (2)

Answer If Please enter the code announced by the experimenters to continue. Text Response Is Equal to MI28

Q108 How many days of the previous week did you practice mindfulness?

Answer If Please enter the code announced by the experimenters to continue. Text Response Is Equal to MI28

Q109 Overall, how many hours did you spend with learning and practicing mindfulness during the previous week?

Answer If Please enter the code announced by the experimenters to continue. Text Response Is Equal to MI28

Q110 Please describe in at least 100 words the mindfulness exercises you did the previous week.

Answer If Please enter the code announced by the experimenters to continue. Text Response Is Equal to MI28

Q111 What did you gain from the mindfulness course during the previous week? Please describe in at least 50 words. Feel free to add critical remarks as well.

Answer If Please enter the code announced by the experimenters to continue. Text Response Is Equal to MI28

Q112 How difficult/easy did you find practising mindfulness during the previous week?

- ❍ Very difficult (1)
- ❍ Difficult (2)
- ❍ Neither difficult nor easy (3)
- ❍ Easy (4)
- ❍ Very easy (5)

Answer If Please enter the code announced by the experimenters to continue. Text Response Is Equal to MI28

Q113 How useful did you find mindfulness for relaxation purposes during the previous week?

- ❍ Very useful (1)
- ❍ Somewhat useful (2)
- ❍ Not useful at all (3)

Answer If Please enter the code announced by the experimenters to continue. Text Response Is Equal to MI28

Q114 Overall, how would you rate the previous week's mindfulness instruction?

- ❍ Excellent (1)
- ❍ Very god (2)
- ❍ Good (3)
- ❍ Fair (4)
- ❍ Poor (5)

Answer If Please enter the code announced by the experimenters to continue. Text Response Is Equal to MI28

Q115 Would you recommend the mindfulness course to a friend?

- Yes (1)
- No (2)

Appendix D: Time preference and revealed preference measures

Question	This Week (£)	Next Week (£)	Question	Next Week (£)	In 2 Weeks (£)
1	3.80	4.00	11	3.80	4.00
2	3.60	4.00	12	3.60	4.00
3	3.40	4.00	13	3.40	4.00
4	3.20	4.00	14	3.20	4.00
5	3.00	4.00	15	3.00	4.00
6	2.80	4.00	16	2.80	4.00
7	2.60	4.00	17	2.60	4.00
8	2.40	4.00	18	2.40	4.00
9	2.20	4.00	19	2.20	4.00
10	2.00	4.00	20	2.00	4.00

Table 2: Time preference measure Session 1

Question	This Week (£)	In 2 Weeks (£)	Question	In 4 Months (£)	In 4 M & 2 Wks (£)
1	30	31	6	30	31
2	30	32	7	30	32
3	30	33	8	30	33
4	30	34	9	30	34
5	30	35	10	30	35

Table 3: Time preference measure Sessions 6 and 7

Scenario	Current Choice	Price (£)
1	Option 1: high calorie	2.60
	Option 2: low calorie	2.00
2	Option 1: high calorie	2.40
	Option 2: low calorie	2.00
3	Option 1: high calorie	2.20
	Option 2: low calorie	2.00
4	Option 1: high calorie	2.00
	Option 2: low calorie	2.00
5	Option 1: high calorie	1.80
	Option 2: low calorie	2.00
6	Option 1: high calorie	1.60
	Option 2: low calorie	2.00
7	Option 1: high calorie	1.40
	Option 2: low calorie	2.00

Table 4: Revealed preference measure

Appendix E: Further results on compliance

Table 5: Effect of MBSR on the frequency of meditation (0-less than once a week to 3-almost every day)

	Coeff.	SE
MBSR	0.178**	0.088
Session 2	0.103	0.063
Session 3	0.060	0.059
Session 4	0.060	0.041
Session 5	0.022	0.047
Session 6	0.012	0.041
Session 7	0.165*	0.090
MBSR & Session 2	0.694***	0.138
MBSR & Session 3	1.076***	0.123
MBSR & Session 4	1.145***	0.122
MBSR & Session 5	1.032***	0.116
MBSR & Session 6	1.212***	0.114
MBSR & Session 7	0.113	0.152
Intercept	0.099	0.369
Individual random effects	Yes	
Control variables	Yes	
No. of individuals	138	

Notes: Robust standard errors; $***p < 0.01, **p < 0.05, *p < 0.1$.

Appendix F: The Impact of MBSR on Perceived Stress Score (PSS) and Anxiety Measures, Sample of Individuals Present at Session 6 or 7

Table 6: The Impact of MBSR on PSS and Anxiety, Individuals Present at Session 6 or 7

	[1]		[2]		[3]	
	PSS		Anxiety Now		Anxiety These Days	
	Coeff.	SE	Coeff.	SE	Coeff.	SE
MBSR	1.057	1.109	1.094**	0.467	0.462	0.462
Session 2	.	.	0.624	0.382	0.298	0.357
Session 3	.	.	1.066***	0.359	0.377	0.304
Session 4	.	.	0.213	0.387	-0.049	0.323
Session 5	.	.	0.457	0.371	-0.073	0.312
Session 6	1.156**	0.505	0.382	0.359	-0.084	0.282
Session 7	2.321***	0.873	0.839**	0.402	0.387	0.365
MBSR & Session 2	.	.	-0.844	0.593	-0.278	0.494
MBSR & Session 3	.	.	-0.926	0.595	-0.557	0.517
MBSR & Session 4	.	.	-0.153	0.576	-0.131	0.484
MBSR & Session 5	.	.	-0.164	0.554	0.130	0.490
MBSR & Session 6	-1.796*	0.951	0.038	0.585	0.184	0.474
MBSR & Session 7	-2.417*	1.344	-0.986	0.690	-0.510	0.604
Intercept	16.162**	7.253	6.546***	2.070	8.145***	2.205
Individual random effects	Yes		Yes		Yes	
Control variables	Yes		Yes		Yes	
No. of individuals	112		112		112	

Notes: Robust standard errors; $***p < 0.01, **p < 0.05, *p < 0.1$.

Appendix G: Summary Statistics of the Stressful Tasks

Table 7: Summary Statistics of the Stressful Tasks

Session 1: Computerised cognitive ability and knowledge test					
	Treatment		Control		Diff
	Mean	SD	Mean	SD	
Task stressful (0-10)	7.373	0.204	7.611	0.166	-0.238
Task not enjoyable (0-10)	6.373	0.284	6.069	0.279	0.304
Task difficult (0-10)	7.179	0.187	6.917	0.192	0.262
Over-confident (0/1)	0.209	0.05	0.155	0.043	0.054

Session 6: Publicly performed cognitive ability and knowledge test					
	Treatment		Control		Diff
	Mean	SD	Mean	SD	
Task stressful (0-10)	6.36	0.282	6.746	0.235	-0.386
Task not enjoyable (0-10)	4.68	0.376	5.206	0.307	-0.526
Task difficult (0-10)	5.16	0.272	5.016	0.234	0.144
Over-confident (0/1)	0	0	0.048	0.027	-0.048

Session 7: Computerised Stroop test					
	Treatment		Control		Diff
	Mean	SD	Mean	SD	
Task stressful (0-10)	6.524	0.311	6.151	0.226	0.373
Task not enjoyable (0-10)	4.762	0.381	4.623	0.285	0.139
Task difficult (0-10)	4.714	0.296	4.642	0.264	0.073
Over-confident (0/1)	0.167	0.063	0.021	0.021	0.145**

, **, * indicate significance levels at 10%, 5% and 1% respectively*

Appendix H: The Impact of MBSR on Health-related Behaviours and Eating Habits

We collect a number of measures related to eating and healthy eating, in particular. First, we construct summary measures of unhealthy food consumption, counting the number of unhealthy items participants report having consumed the previous day (from a list we provided; see Appendix C for details), and using the binary indicators of avoiding foods high in fat or cholesterol, and trying to eat foods high in fibre. Second, we focus on measures related to "emotional" or "comfort eating" based on the Compulsive Eating Scale (**?**).

Next, we have measures (based on self-reports) of the frequency of smoking and alcohol consumption, as well as the average number of hours slept. In Session 1, the respondents were asked generally about their smoking and drinking habits, while, in the other sessions, the questions referred to the previous week. The detailed weekly questions can be found in Appendix C.

We also collected a measure of preferences for "healthy foods", using a revealed preference approach. Participants were asked to make a real choice between a high-calorie and a low-calorie option. Each option is a combination of a snack and a drink. Participants were first asked to choose sequentially among three high-calorie snacks, three high-calorie drinks, three low-calorie snacks and three low-calorie drinks. We then constructed a low-calorie option by combining their preferred low-calorie snack with their preferred low-calorie drink, and a high-calorie option by combining their preferred high-calorie drink and high-calorie snack. Participants were endowed with £4 and asked to pick between the high- and low-calorie options, each of them associated with different prices. The options are listed Appendix D. The price of the chosen item would be deducted from the £4 endowment. They were asked to choose between their preferred high- and low-calorie options at different prices.[1]

We construct a measure of preference for the low-calorie option, which corresponds to the number of times participants choose that option rather than the high-calorie option.

[1] We also separately asked participants to make decisions involving receiving the snack and drink immediately, but paying later. Unfortunately, these measures cannot be used in the analysis due to an error in programming.

Table 8: The Impact of MBSR on Health-related Behaviours

	Average hours of sleep/day		Smoking		Frequency of alcohol consumption	
	Coeff.	SE	Coeff.	SE	Coeff.	SE
MBSR	-0.028	0.192	0.025	0.120	0.180	0.130
Session 2	0.055	0.347	0.038	0.033	0.052	0.109
Session 3	-0.264**	0.112	-0.049	0.057	0.200*	0.108
Session 4	-0.176*	0.098	-0.049	0.061	0.479***	0.111
Session 5	-0.241**	0.121	-0.081	0.052	0.355***	0.120
Session 6	-0.583***	0.143	-0.002	0.044	-0.195**	0.098
Session 7	0.029	0.487	0.047	0.051	-0.295***	0.084
MBSR S2	1.436	1.205	0.040	0.064	0.076	0.159
MBSR S3	0.049	0.191	0.065	0.071	0.177	0.150
MBSR S4	-0.325*	0.185	0.086	0.073	-0.171	0.167
MBSR S5	-0.214	0.223	0.071	0.070	-0.179	0.182
MBSR S6	0.140	0.198	0.002	0.069	0.096	0.138
MBSR S7	-0.378	0.520	-0.044	0.090	0.072	0.127
Intercept	9.452***	1.578	1.513***	0.490	5.003***	1.009
Ind. RE	Yes		Yes		Yes	
Control var.	Yes		Yes		Yes	
No. of ind.	138		138		138	

, **, * indicate significance levels at 10%, 5% and 1% respectively*

Table 9: The Impact of MBSR on eating habits measured only at sessions 1, 6 and 7

	# of low-calorie options chosen, 0 to 10		Avoid fat		Eat high fibre	
	Coeff.	SE	Coeff.	SE	Coeff.	SE
MBSR	0.596	0.714	-0.04	0.087	-0.082	0.083
Session 6	-1.812***	0.420	0.079*	0.048	-0.046	0.058
Session 7	-1.451***	0.471	-0.005	0.064	-0.1	0.069
MBSR S6	-1.008	0.671	-0.005	0.067	0.014	0.09
MBSR S7	-0.994	0.737	0.08	0.08	0.135	0.093
Intercept	3.980	2.482	1.848***	0.455	1.573***	0.412
Ind. RE	Yes		Yes		Yes	
Control var.	Yes		Yes		Yes	
No. of ind.	132		138		138	

, **, * indicate significance levels at 10%, 5% and 1% respectively*

Table 10: The Impact of MBSR on Eating Habits

	Unhealthy food items yesterday		Out of control with food		Eat because upset, nervous		Eat regular (1 always to 4 never)		Snack, studying (0 never to 4 always)		Eat more while studying (0 to 4)		Eat because bored (0 to 4)		Eat much fast (0 to 4)	
	Coeff.	SE	Coeff.	SE	Coeff.	SE	Coeff.	SE	Coeff.	SE	Coeff.	SE	Coeff.	SE	Coeff.	SE
MBSR	-0.267	0.593	0.126	0.156	0.086	0.176	0.028	0.14	0.17	0.244	-0.102	0.25	-0.21	0.168	0.161	0.18
Session 2	-0.245	0.429	-0.274***	0.101	-0.386***	0.139	-0.086	0.079	-0.145	0.179	-0.441**	0.183	-0.516***	0.144	-0.197*	0.111
Session 3	-0.152	0.389	-0.275**	0.111	-0.431***	0.138	-0.166*	0.096	-0.269	0.176	-0.617***	0.179	-0.596***	0.133	-0.373***	0.102
Session 4	0.142	0.496	-0.246**	0.123	-0.549***	0.135	-0.078	0.088	-0.401**	0.157	-0.793***	0.158	-0.758***	0.129	-0.270**	0.118
Session 5	0.127	0.455	-0.306***	0.115	-0.530***	0.143	-0.208**	0.085	-0.325**	0.158	-0.702***	0.182	-0.752***	0.135	-0.294**	0.119
Session 6	-0.236	0.492	-0.16	0.108	-0.379***	0.14	-0.183*	0.098	-0.125	0.167	-0.432**	0.171	-0.616***	0.126	-0.216*	0.119
Session 7	-0.226	0.55	0.015	0.131	0.076	0.145	-0.051	0.106	.		.		-0.336**	0.144	-0.185	0.136
MBSR S2	0.355	0.588	0.167	-0.007	0.178	-0.066	0.124	0.206	0.269	0.391	0.272	-0.128	0	0.179	-0.133	0.167
MBSR S3	0.235	0.501	0.035	0.186	0.025	0.195	0.04	0.14	0.101	0.25	0.214	0.257	0.232	0.182	0.015	0.176
MBSR S4	-0.246	0.618	-0.223	0.185	-0.041	0.191	-0.078	0.136	0.234	0.252	0.385*	0.227	0.241	0.185	0.058	0.168
MBSR S5	-0.405	0.559	-0.045	0.168	0.057	0.202	0.143	0.138	-0.149	0.261	0.354	0.254	0.201	0.196	-0.021	0.166
MBSR S6	-0.488	0.647	-0.195	0.162	-0.038	0.191	0.145	0.144	-0.101	0.26	0.198	0.246	0.084	0.189	-0.142	0.191
MBSR S7	-0.791	0.7	-0.094	0.207	-0.417*	0.228	-0.056	0.177	.		.		0.222	0.222	0.086	0.202
Intercept	5.910*	3.201	1.458**	0.688	0.628	0.78	1.868**	0.9	1.269	0.935	1.485	1.065	0.222	0.962	1.714**	0.723
Ind. RE	Yes		Yes		Yes		Yes		Yes		Yes		Yes		Yes	
Control var.	Yes		Yes		Yes		Yes		Yes		Yes		Yes		Yes	
No. of ind.	138		138		138		138		134		134		138		138	

, **, * indicate significance levels at 10%, 5% and 1% respectively*
