# Supplementary material for: Mind training, stress and behaviour—A randomised experiment
Source: PLoS One. 2021 Nov 12;16(11):e0258172. doi: 10.1371/journal.pone.0258172 (PMC8589216; doi:10.1371/journal.pone.0258172)
Supplement: Attachment — Submitted filename: Response to reviewers 3 MB.pdf [file pone.0258172.s002.pdf]

Reviewers' comments:

Reviewer's Responses to Questions

Comments to the Author

1. Is the manuscript technically sound, and do the data support the conclusions?

The manuscript must describe a technically sound piece of scientific research with data that supports the conclusions. Experiments must have been conducted rigorously, with appropriate controls, replication, and sample sizes. The conclusions must be drawn appropriately based on the data presented.

Reviewer #1: Yes

Reviewer #2: Yes

---

2. Has the statistical analysis been performed appropriately and rigorously?

Reviewer #1: Yes

Reviewer #2: Yes

---

3. Have the authors made all data underlying the findings in their manuscript fully available?

The PLOS Data policy requires authors to make all data underlying the findings described in their manuscript fully available without restriction, with rare exception (please refer to the Data Availability Statement in the manuscript PDF file). The data should be provided as part of the manuscript or its supporting information, or deposited to a public repository. For example, in addition to summary statistics, the data points behind means, medians and variance measures should be available. If there are restrictions on publicly sharing data—e.g. participant privacy or use of data from a third party—those must be specified.

Reviewer #1: Yes

Reviewer #2: Yes

---

4. Is the manuscript presented in an intelligible fashion and written in standard English?

PLOS ONE does not copyedit accepted manuscripts, so the language in submitted articles must be clear, correct, and unambiguous. Any typographical or grammatical errors should be corrected at revision, so please note any specific errors here.

Reviewer #1: Yes

Reviewer #2: Yes

---

5. Review Comments to the Author

Please use the space provided to explain your answers to the questions above. You may also include additional comments for the author, including concerns about dual publication, research ethics, or publication ethics. (Please upload your review as an attachment if it exceeds 20,000 characters)

Reviewer #1: This is an interesting study. I like it that the authors have measured stress in different ways and thus replicated prior results with a non-self selected sample.
My only concern is whether the lack of results in the decision-making tasks is related to the sample size. I would like the authors to discuss what effect sizes their sample are powered to detect.

Response from the authors: Thank you for your positive view on the paper. Your point about lack of power is well taken. Power calculations were not common at the time we conducted the experiment, and our understanding is that ex-post power calculations are problematic (see for example this discussion on a World Bank Blog. In our view, it may make sense to perform power calculations for analyses of outcomes that have been previously studied in the literature. For risk, we can base our calculations on the study of Crosetto & Filippin (2013). In their static version of the game, they report a mean of 46.5 boxes collected and a standard deviation of 15.1. With this mean and standard deviation, we were powered to detect an effect size of 15% (going from 46 to 53 boxes) with power 0.80 and significance at 5%.

For the estimates of impatience and present bias, it is harder to find studies that match exactly out outcome variables (other studies have different incentives, so we cannot assume that they provide good estimates of the fraction of present biased individuals, and the discount rates we estimate are also specific to the timelines we used.) So for these variables, we would be reluctant to present ex-post power calculations. We have chosen to acknowledge more explicitly the possibility that the lack of significant results may be due to small sample size.

Reviewer #2: I liked the paper very much. Its clear and very well executed. As authors know they have a very reduced sample but, on the other hand this is a novel idea.

- Table 2: I feel that is better to say "risk taking" rather than "# boxes collected (BRET)" --> Compare Table 2 and 4

Response from authors: Thank you for the suggestion, which we have followed.

- perhaps the number of table might be reduced (some of the might be merged)

Response from authors: We agree and we have merged Tables 8 and 9 from the previous version

Additional references to consider:

- On time preferences: https://mpra.ub.uni-muenchen.de/103660/

Response from authors: This is an interesting paper but since we are not using hypothetical questions, we are not too sure where to fit the reference in. Also, the reference list is on the long side already, so we are reluctant to make it even longer.

---

6. PLOS authors have the option to publish the peer review history of their article (what does this mean?). If published, this will include your full peer review and any attached files.

If you choose "no", your identity will remain anonymous but your review may still be made public.

Do you want your identity to be public for this peer review? For information about this choice, including consent withdrawal, please see our Privacy Policy.

Reviewer #1: No

Reviewer #2: No

[NOTE: If reviewer comments were submitted as an attachment file, they will be attached to this email and accessible via the submission site. Please log into your account, locate the manuscript record, and check for the action link "View Attachments". If this link does not appear, there are no attachment files.]

While revising your submission, please upload your figure files to the Preflight Analysis and Conversion Engine (PACE) digital diagnostic tool, https://pacev2.apexcovantage.com/. PACE helps ensure that figures meet PLOS requirements. To use PACE, you must first register as a user. Registration is free. Then, login and navigate to the UPLOAD tab, where you will find detailed instructions on how to use the tool. If you encounter any issues or have any questions when using PACE, please email PLOS at figures@plos.org. Please note that Supporting Information files do not need this step.
